# Case Report: Multifactorial Intervention for Safe Aging in Place

**DOI:** 10.3390/geriatrics10030068

**Published:** 2025-05-20

**Authors:** Ashwini Kulkarni

**Affiliations:** School of Rehabilitation Sciences, Ellmer College of Health Sciences, Macon and Joan Brock Virginia Health Sciences, Old Dominion University, Norfolk, VA 23529, USA; a1kulkar@odu.edu; Tel.: +1-765-772-5050

**Keywords:** falls prevention, multidisciplinary intervention, aging in place, geriatric rehabilitation, balance and mobility, chronic conditions and fall risk

## Abstract

**Background/Objectives**: Falls are a leading cause of morbidity in older adults, particularly those with multiple comorbidities. A multidisciplinary approach addressing physical, psychological, and environmental factors is essential for reducing fall risk and supporting aging in place. This report evaluates the effectiveness of a multidisciplinary, multifactorial approach in managing high fall risk in an older adult with diabetes, hypertension, and osteoporosis. **Methods**: A 72-year-old woman with a recurrent history of falls participated in an 8-week intervention as part of the American Physical Therapy Association (APTA) balance and falls prevention credential program. This study was conducted in Virginia Beach, USA, at the participant’s residence. A single-subject design investigation was conducted, measuring outcomes including the Balance Evaluation Systems Test (BESTest), gait speed, Timed Up and Go (TUG), fear of falling, and balance confidence at baseline and post-intervention. **Results:** The participant had impaired baseline values across various variables and was classified as a recurrent high-risk faller. After 8 weeks of intervention, clinically meaningful improvements with large effect sizes were observed: self-selected gait speed improved by 25%, BESTest scores improved by 50%, Falls Efficacy—International (FES I) scores improved by 26%, and Activity Balance Confidence (ABC) scores improved by 26%. No falls or adverse events occurred during the intervention period, and the patient reported enhanced mobility and safety at home. **Conclusions**: A tailored multidisciplinary approach effectively addressed the physical, psychological, and environmental factors contributing to high fall risk. This highlights the importance of patient-centered interventions in managing fall risk and promoting safe aging in place. Continued education, environmental adaptations, and regular follow-up are essential for long-term fall prevention.

## 1. Introduction

Falls are a major public health problem [1,2,3,4] and one of the most significant threats to older adults’ ability to safely age in place [5,6]. Among community-dwelling older adults, annual fall rates range from 32% to 42% (World Health Organization [WHO], 2021). The consequences of falling can be devastating—physically, psychologically, and economically—often leading to a cascade of events including injury, hospitalization, loss of independence, and institutionalization [7,8,9]. The economic burden is equally concerning, with fall-related healthcare costs exceeding $50 billion annually in the United States alone (Centers for Disease Control and Prevention [CDC], 2023).

The risk of falling is rarely attributable to a single factor. Instead, it stems from a complex interplay of intrinsic factors—such as decreased strength, impaired balance, polypharmacy, and chronic health conditions—and extrinsic factors like environmental hazards, poor lighting, and unsafe home design [10,11,12,13]. These multifactorial risks are further compounded in older adults with multiple comorbidities, making fall prevention both a clinical necessity and a public health priority.

Given these complexities, a shift toward individualized, multidisciplinary interventions is critical. Integrating physical, psychological, environmental, and behavioral strategies has the potential to more effectively manage fall risk and support safe aging in place. Emerging models of care now emphasize sustainable, culturally sensitive, and patient-centered approaches that reflect the biopsychosocial realities of aging [14,15].

Supporting independent living—commonly referred to as aging in place—has become a central goal in geriatric care [16,17,18]. Nearly 87% of older adults express a strong preference to remain in their homes as they age (American Association of Retired Persons [AARP], 2023) [19]. However, this goal is often challenged by the presence of chronic conditions, frailty, and functional limitations. Falls represent a key barrier to this objective, frequently triggering functional decline, fear of falling, and a subsequent cycle of inactivity and dependence.

To support independence, research underscores the need for comprehensive, interdisciplinary approaches that extend beyond isolated interventions. While traditional fall prevention strategies—such as exercise programs [20,21] or home modifications [22,23]—may be effective for some, they often fall short in addressing the complex needs of older adults with multiple comorbidities. In this context, conditions like diabetes, hypertension, and osteoporosis introduce specific physiological and pharmacological challenges that require nuanced, multifaceted care. For example, diabetic peripheral neuropathy can impair proprioception and balance [24], while orthostatic hypotension associated with cardiovascular disease [25] can interfere with mobility and rehabilitation efforts. Moreover, the psychological consequences of falls, including fear of falling [26,27,28], further compound physical limitations and contribute to functional decline.

This case report presents the implementation of a multidisciplinary, multifactorial intervention for fall prevention in an older adult with diabetes, hypertension, and osteoporosis. By incorporating physical therapy, behavioral strategies, environmental modifications, and psychological support, this case illustrates a comprehensive model for addressing fall risk. Standardized outcome measures—including the Balance Evaluation Systems Test (BESTest) [29], gait speed [30], Timed Up and Go (TUG) [31], Falls Efficacy Scale—International (FES-I) [32], and Activity Balance Confidence (ABC Scale) [33]—provide objective data to evaluate the intervention’s impact. Rather than relying on short-term or single-focus strategies, this report emphasizes the importance of sustainable, integrated care models that support long-term safety and autonomy. The intervention described was delivered in a Virginia Beach, USA, home health setting, with an interdisciplinary team approach reflective of current best practices in geriatric rehabilitation. Ultimately, it contributes to the growing recognition that effective fall prevention requires personalized, interdisciplinary care tailored to the unique needs of each older adult.

## 2. Methods

This unblinded, single-case study was conducted in July 2024 as part of the American Physical Therapy Association (APTA) Balance and Falls Prevention Credential program in Portland, Oregon. This single-subject design was chosen to illustrate how a personalized, multidisciplinary intervention can be effectively implemented in a real-world clinical context where randomized trials may not be feasible. This study adhered to the ethical principles outlined in the Declaration of Helsinki. Written informed consent was obtained from the participant, and all procedures followed Health Insurance Portability and Accountability Act (HIPAA) guidelines to ensure data privacy and participant confidentiality.

This case report highlights the complex interplay of physical, psychological, and environmental risk factors influencing an older adult’s ability to age safely in place—a topic of growing public health importance as the aging population increases. The featured patient is a 72-year-old Asian female residing independently in a single-story ranch-style home. She was referred to physical therapy due to a high fall risk, declining balance, and gait instability. Her chief complaints included unsteadiness, fear of falling, and difficulty rising from the floor after gardening—factors that significantly impacted her confidence and participation in daily activities.

Her fall history is concerning, with over five documented falls in the past six months and two recent near-fall episodes—one in the bathroom and another at the front entrance of her home. Her medical profile includes multiple chronic conditions such as hypertension (diagnosed 15 years ago), hyperlipidemia, type II diabetes (managed with an insulin pump for five years), low bone density (DEXA T-score: −3.5; obtained from medical reports), and a conservatively managed wrist fracture. The patient took the following medications to manage their comorbidities: lisinopril and amlodipine for managing hypertension, atorvastatin for hyperlipidemia, and an insulin pump was used for delivering insulin two times a day with a total daily dose of 40 units to manage type II diabetes. Osteoporosis was treated with diet and supplements (calcium, vitamin D supplements, and bisphosphonates (Actonel)). She also reports poor sleep quality and regularly (one pill every night) uses Alprax as a sleep aid.

Socially, the patient remains well-connected. Though her children live out of state (Texas and London), she engages in her community by attending Bingo games bi-monthly and a weekly book club. Despite no longer driving, she uses grocery delivery services and continues gardening. Cognitively, she is independent and adept with technology, using MediChart and Alexa for medication and reminders.

Her only prior experience with physical therapy was limited to outpatient rehabilitation following a wrist fracture, which focused primarily on flexibility and strengthening exercises for the wrist. Her current therapy goals include regaining confidence in walking independently with friends and performing household activities more safely—especially the ability to get up from the floor after gardening. The assessment and intervention described in this study were conducted at the patient’s residence.

This case underscores the critical role of multidisciplinary, personalized interventions in supporting older adults’ goals to age in place while reducing fall risk and promoting functional independence.

### 2.1. Assessment

Given the critical importance of identifying modifiable risk factors to support safe aging in place, a comprehensive clinical assessment was conducted (Table 1). These findings underscored the need for a multidisciplinary intervention approach tailored to the patient’s complex needs and environment.

To ensure the accurate evaluation of fall risk, mobility, balance, and psychological readiness, outcome measures were selected based on their proven validity and reliability in older adults. The Timed Up and Go (TUG) Test is a well-established tool for identifying mobility limitations and fall risk [31,34,35]. The Falls Efficacy Scale—International (FES-I) provides a reliable measure of fear of falling and has demonstrated excellent psychometric properties across older populations [36,37,38]. The Balance Evaluation Systems Test (BESTest), including its six subdomains, offers a detailed analysis of postural control deficits and is highly appropriate for individuals with complex, multifactorial impairments [29,39,40]. Gait speed [30,41] and the 30-Second Sit-to-Stand Test [42] are efficient, evidence-based indicators of functional performance and fall risk. The Activities-Specific Balance Confidence (ABC) Scale complements the FES-I by assessing self-perceived balance confidence during daily tasks [33,43]. Additional tools—such as the Four-Stage Balance Test, stair navigation, floor transfer ability, blood pressure and vision screenings, home safety assessment, and lower extremity strength testing—were chosen based on their clinical utility and alignment with CDC STEADI guidelines (CDC). These measures collectively enabled a comprehensive, multifactorial assessment to guide targeted rehabilitation interventions.

In this case, a comprehensive multidisciplinary assessment revealed several contributing factors to fall risk and mobility impairment, underscoring the complexity and importance of individualized interventions to support safe aging in place. Key clinical findings included an orthostatic blood pressure drop of 22 mmHg systolic, which provoked dizziness, as well as near vision impairment that adversely affected balance. Psychological components—specifically, a moderate fear of falling (FES-I score: 35/64) and reduced balance confidence (ABC Scale score: 65%)—further contributed to activity avoidance and potential physical deconditioning. Environmental hazards within her home environment compounded these risks. Although her osteoporosis (DEXA score: −3.5) was not a direct cause of falls, it significantly heightened the risk of injury in the event of one.

**Figure 1:**
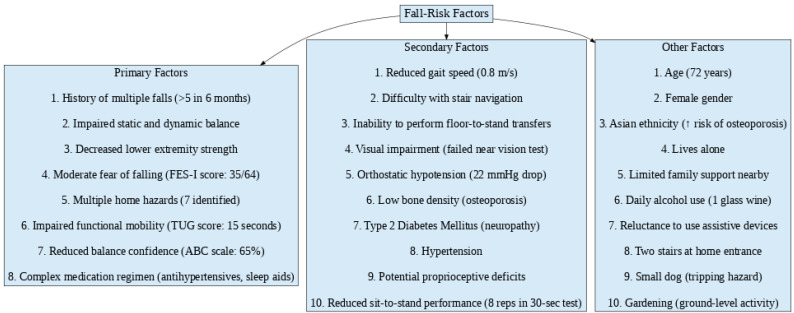
Flowchart depicting the classification of fall-risk factors into primary, secondary, and other categories.

Several challenges complicated the diagnostic process, including the patient’s initial resistance to using assistive devices, limited financial resources that prevented advanced vestibular testing, and delays in implementing a home safety evaluation. Despite these barriers, her outlook remained positive. Addressing psychosocial factors such as fear of falling, alongside home modifications, was critical in enhancing both her safety and mobility. To guide intervention planning, I categorized the contributing factors into primary, secondary, and other influences (Figure 1). Primary factors directly impaired balance, strength, and mobility; secondary factors aggravated these deficits; and other contextual factors—such as age, medical history, and lifestyle—played an indirect yet influential role.

As this is a single-case study, the findings are not intended to be generalized but instead provide valuable insights for customizing interventions in older adults with comparable clinical profiles.

### 2.2. Eight-Week Exercise Intervention Summary

**Figure 2:**
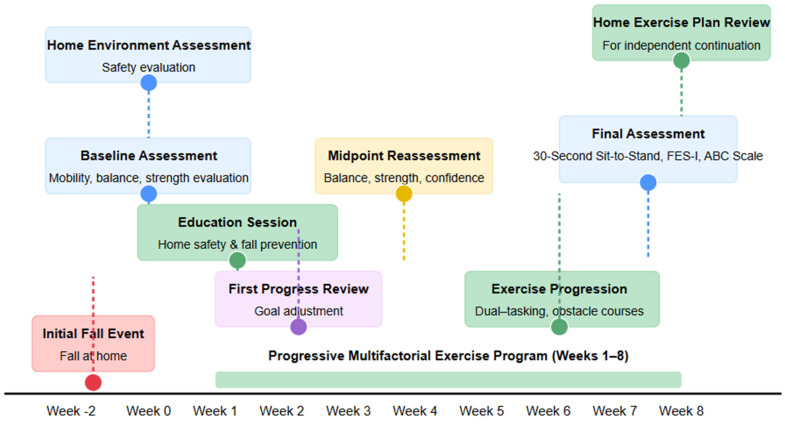
Case timeline: Multifactorial Fall Prevention Intervention.

As part of a comprehensive strategy to promote safe aging in place, the patient participated in an 8-week, evidence-based rehabilitation program designed to enhance lower limb strength, balance, mobility, and confidence while reducing fall risk. The timeline for this study is depicted below (Figure 2).

**Figure 3:**
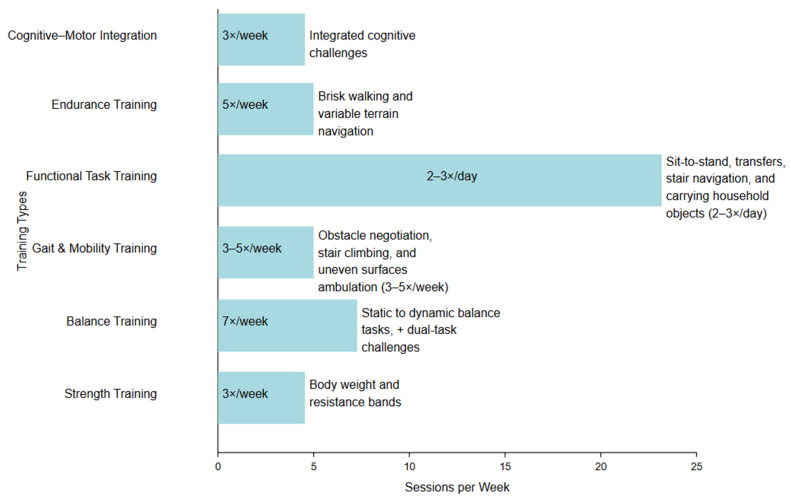
Illustration of intervention components.

Grounded in the FITT (Frequency, Intensity, Time, Type) principles, the program progressively increased in intensity, duration, and complexity to ensure functional carryover into daily life (Figure 3).

Beyond physical training, the program emphasized habit formation, caregiver involvement, and home environment modifications to support long-term adherence and safety. Psychosocial factors were also addressed through confidence-building strategies, gradual exposure to challenging tasks, and community-based engagement opportunities.

### 2.3. Patient Participation in the Therapeutic Plan

Therapeutic goals were jointly defined with the patient at the outset of the program, ensuring alignment with her personal preferences, daily activity priorities, and individual values. The physical therapist engaged the patient in shared decision-making, discussing exercise options and expected outcomes. Goals and exercises were reviewed every two weeks during follow-up calls and sessions, allowing for adjustments based on the patient’s self-reported progress, feedback, and evolving functional needs.

This evidence-based [44,45,46], multifactorial approach fosters lasting mobility improvements and fall prevention, ensuring safe, independent, and active living. The significance of this case lies not only in the clinical outcomes but also in demonstrating the feasibility and impact of such interventions within a real-world context (Table 2).

Intervention adherence was monitored weekly via activity logs that the patient maintained, where they checked a box if they performed exercises as planned.

### 2.4. Psychological and Educational Approach

To address the fear of falling and build confidence, the physical therapist incorporated gradual exposure to progressively challenging tasks, positive reinforcement for task completion, and education on fall prevention strategies. Cognitive behavioral techniques, such as reframing negative thoughts about falling and encouraging self-efficacy through goal setting, were integrated into sessions. Patients were also taught to recognize and celebrate small improvements to promote a positive mindset toward mobility.

## 3. Results

All assessment measures were scored and interpreted against clinically established normative values, and meaningful changes were evaluated using Minimal Clinically Important Differences (MCID) and Cohen’s *d* effect sizes.

Over the course of an eight-week intervention, the patient demonstrated substantial and clinically significant improvements in mobility, balance, lower-body strength, and confidence. As shown in Figure 3, improvements ranging from 25 to 75% were observed across multiple assessment measures. These improvements translated into a notable reduction in fall risk and enhanced functional independence, directly supporting the goals of aging in place safely (Figure 3).

**Figure 4:**
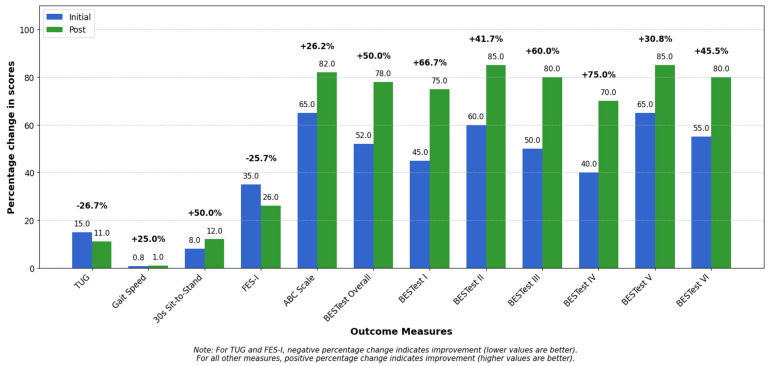
Initial and post-intervention scores across clinical outcome measures. Bar graph showing initial (blue) and post-intervention (green) scores across twelve functional mobility and balance assessment measures. Values displayed within each bar represent raw scores, while percentages above each pair indicate relative change between assessments. For the Timed Up and Go (TUG) and Falls Efficacy Scale (FES-I), negative percentage changes represent improvement as lower scores indicate better performance. For all other measures (Gait Speed, 30s Sit-to-Stand, ABC Scale, and BESTest components), positive percentage changes represent improvement.

At baseline, the Timed Up and Go (TUG) test revealed elevated fall risk, with a completion time of 15 s. By the end of the program, this improved to 11 s—a 26.67% improvement as indicated in Figure 3, surpassing the MCID threshold of 3 s (*d* = 1.6, Table 3)—indicating a meaningful enhancement in functional mobility and safety. Likewise, gait speed improved from 0.8 m/s to 1.0 m/s, representing a 25% gain per Figure 4 exceeding the MCID of 0.1 m/s (*d* = 1.3, Table 3), consistent with thresholds for safe community ambulation.

Functional strength improvements were evident in the 30-Second Sit-to-Stand test, where repetitions increased from 8 to 12—the 50% improvement visualized in Figure 4 (*d* = 2.0, Table 3) and beyond the MCID of 2 repetitions—reflecting better lower-limb strength and endurance. This gain reflects meaningful enhancements in lower-limb strength and endurance, translating into an improved ability to perform essential daily activities such as rising from a chair, transferring, and maintaining independence.

Significant gains in balance were also observed. Initially, the patient was unable to maintain a tandem stance during the Four Stage Balance Test, but by week eight, could sustain it for 10 s, indicating enhanced static balance. The Balance Evaluation Systems Test (BESTest) score increased from 52% to 78%, as reflected in the 50% improvement in BESTest overall shown in Figure 4, exceeding the MCID of 10% (*d* = 4.0, Table 3), suggesting notable improvements in postural control, anticipatory adjustments, and reactive balance responses.

Confidence in mobility—a key factor for maintaining independence—also improved. The Activities-Specific Balance Confidence (ABC) Scale rose from 65% to 82%, a 26.15% improvement clearly depicted in Figure 4, surpassing the 14% MCID (*d* = 2.43, Table 3). Meanwhile, the Falls Efficacy Scale—International (FES-I) score improved from 35 to 26, demonstrating the 25.71% positive change illustrated in Figure 4, exceeding the MCID of 6 points (*d* = 2.25, Table 3), indicating reduced fear of falling—a critical barrier to physical activity and participation.

Functionally, the patient progressed from requiring full handrail support for stair navigation to using minimal assistance, demonstrating increased strength and coordination. A key milestone was the ability to independently perform a floor-to-stand transfer, aligning with the substantial BESTest subsection improvements of 41–75% shown in Figure 4, reflecting a meaningful recovery in strength, mobility, and self-efficacy.

Beyond physical outcomes, the patient demonstrated high adherence (85%) to their home exercise program and implemented targeted home safety modifications, reducing environmental fall hazards from seven to two. These changes created a safer home environment, further supporting independent living.

Overall, the intervention led to clinically meaningful gains across all domains, with Figure 4 providing visual confirmation of improvements in every assessment measure, with large effect sizes reinforcing the magnitude of improvement. This case underscores the powerful role of multidisciplinary rehabilitation in promoting strength, stability, and confidence—cornerstones of safe aging in place.

## 4. Discussion

This case study demonstrates the effectiveness of a multidisciplinary, multifactorial intervention in reducing fall risk and improving mobility, balance, strength, and confidence in an older adult with multiple comorbidities. The observed improvements across standardized outcome measures reinforce the growing evidence that individualized, comprehensive fall prevention programs are essential for managing the complex interplay of intrinsic and extrinsic fall risk factors.

### 4.1. Linking Results to Fall Prevention and Aging in Place

The patient’s gains in mobility, balance confidence, and gait performance highlight the benefits of an interdisciplinary intervention.

The improvement in functional mobility and gait speed—evidenced by the reduction in TUG time (15 s to 11 s, MCID = 3 s) and an increase in gait speed (0.8 m/s to 1.0 m/s, MCID = 0.1 m/s)—can be attributed to the physical rehabilitation component, which focused on balance and strength training. This is because strengthening key muscle groups, particularly in the lower limbs, improves stability and helps the body better handle destabilizing forces (e.g., uneven surfaces or quick movements) [57,58,59]. Additionally, balance training improves postural control, reducing fall risk by enhancing the body’s ability to stay upright and recover from slight imbalances.

The BESTest score increase (52% to 78%, MCID = 10%) suggests enhanced postural control, reducing fall risk. This improvement reflects the intervention’s success in targeting balance deficits, which are a common issue in older adults, particularly those with comorbidities such as diabetes and osteoporosis. Improved static balance in the Four Stage Balance Test and tandem stance further supports fall prevention in individuals with these health conditions.

### 4.2. The Role of Psychological and Behavioral Factors

Fear of falling contributes to inactivity, deconditioning, and social isolation. The improvement in FES-I (35 to 26, MCID = 6) and ABC Scale (65% to 82%) reflects increased confidence and reduced anxiety. Confidence-building exercises and gradual exposure to balance challenges played a key role in overcoming avoidance behaviors and promoting physical activity [60,61]. These strategies align with Bandura’s self-efficacy theory, emphasizing mastery experiences and positive reinforcement to build confidence in mobility. Moreover, by addressing psychological barriers such as fear of falling, the intervention supported principles of active aging—encouraging autonomy, participation, and quality of life through enhanced physical and emotional resilience. By addressing psychological barriers like fear of falling, the intervention helped the patient become more active, which in turn supported their physical rehabilitation progress.

### 4.3. Individualized, Multifactorial Approaches vs. Single-Intervention Models

This study stands out due to its individualized, multifactorial approach, integrating strength, balance, gait, and cognitive components tailored to the patient’s specific needs and progress. Unlike single-intervention models that focus on one aspect of rehabilitation, this approach recognizes the complexity of functional decline in older adults and addresses multiple factors simultaneously. Furthermore, the patient population in this study—older adults with comorbidities such as frailty, muscle weakness, and balance impairments—represents a group that is underexplored in the literature. Most interventions tend to focus on isolated conditions, but our study provides a holistic framework that accounts for the interplay between various factors that affect mobility and independence in older adults. By combining strength training with cognitive challenges and tailored psychosocial support, this intervention provides a more comprehensive and realistic approach to rehabilitation, enhancing both physical and emotional resilience.

This case study demonstrates the effectiveness of a multifactorial approach integrating the following:

- Physical rehabilitation (balance and strength training)
- Behavioral strategies (confidence-building, fear reduction)
- Environmental modifications (home safety adaptations)
- Patient education (structured home exercise programs)

By integrating these elements, the intervention addressed both intrinsic factors (e.g., muscle strength, balance, and fear of falling) and extrinsic factors (e.g., home hazards and social support) that contribute to fall risk. This approach is in line with research advocating interdisciplinary fall prevention for individuals with complex health conditions, including diabetes-related proprioception deficits and osteoporosis-related fracture risks. Furthermore, addressing polypharmacy helped mitigate medication-induced fall risk, which may have been an important factor in the observed improvements.

The home exercise plan incorporated functional strength training exercises like sit-to-stand practice, seated leg extensions, and step-ups to improve lower limb power necessary for daily activities. Balance training included static standing with feet together, single-leg stance, tandem walking, and obstacle course navigation to enhance postural control and dynamic stability. Behavioral strategies such as gradual exposure to challenging balance tasks and positive reinforcement were used to build confidence and reduce fear of falling. Environmental modifications included removing trip hazards, improving lighting, and installing grab bars to create a safer home environment. Patient education focused on teaching proper exercise technique, developing a daily routine, and encouraging self-monitoring through an activity log and telemonitoring calls. Together, these elements addressed muscle strength, balance deficits, psychological barriers, and environmental risks contributing to falls.

### 4.4. Sustainability and Long-Term Fall Prevention

Sustained adherence is key to long-term success. The 85% adherence rate to home exercises highlights the role of patient engagement and self-management. Research emphasizes that structured follow-ups and caregiver involvement improve long-term fall prevention outcomes. Regular appointments with a physiotherapist may help maintain these functional gains over time, providing continued guidance and progression of exercises as needed. Environmental modifications, reducing household fall hazards from seven to two, further support aging in place. Given that most falls occur at home, these changes—combined with improved physical function—enhance safety and reduce reliance on institutional care. The reduction in home hazards directly contributed to the patient’s confidence and safety, reinforcing the physical improvements gained through rehabilitation. Furthermore, the involvement of the general practitioner or family doctor in referring patients to appropriate rehabilitation programs and reviewing medications to avoid unnecessary polypharmacy is essential in a holistic fall prevention strategy. By improving mobility, reducing fall-related anxiety, and promoting safe, independent living, interdisciplinary fall prevention programs align with value-based care models, reducing hospitalizations and long-term care admissions.

### 4.5. Limitations

While this case study highlights the effectiveness of a multifactorial fall prevention intervention, several limitations must be considered. As a single-case study, its findings may not be generalizable to broader populations with varying comorbidities and functional levels. The lack of long-term follow-up limits understanding of sustained benefits, while self-reported measures introduce potential bias. Additionally, external factors such as medication changes and social support may have influenced outcomes. Variability in home environments and access to resources further impacts intervention feasibility. Despite these limitations, this case study provides valuable insights into the real-world application of fall prevention strategies in older adults with complex health conditions, offering a foundation for future research and the development of personalized, multifaceted interventions for fall risk reduction. Future research should explore larger, diverse samples, objective assessments, and long-term adherence to ensure broader applicability of fall prevention strategies.

## 5. Conclusions

This case study demonstrates the success of a multifactorial, interdisciplinary approach in reducing fall risk and improving functional independence in an older adult with diabetes, hypertension, and osteoporosis. The significant improvements in mobility, balance, confidence, and independence highlight the importance of personalized, holistic interventions in geriatric rehabilitation. To effectively prevent falls and promote aging in place, fall prevention programs should integrate physical, psychological, and environmental strategies, ultimately reducing healthcare costs and enhancing overall well-being.

## Figures and Tables

**Table 1:** Assessment Outcomes, Identified Impairments, and Rehabilitation Goals. This table summarizes the standardized assessments used to evaluate Mrs. XX’s functional mobility, balance, strength, and fall risk. It details test results, identified impairments, and targeted rehabilitation goals, both short-term (STG) and long-term (LTG). Goals focus on improving balance, mobility, strength, and confidence while addressing key impairments such as postural control, gait stability, fear of falling, and environmental risk factors.

Outcome Measure	Interpretation	Short Term Goals (STGs) (Week 3/8)	Long Term Goals (LTGs) (Week 8/8)	Impairments
Timed Up and Go (TUG) Test	High fall risk; impaired functional mobility	Decrease TUG time to 13 s	Achieve TUG time of 11 s	Balance, gait speed, functional mobility
Falls Efficacy Scale—International (FES-I)	Moderate fear of falling affecting daily activities	Reduce FES-I score to 30/64	Achieve FES-I score of 25/64 or less	Fear of falling, activity avoidance
Four Stage Balance Test	Poor static balance, increasing fall risk	Hold tandem stance for 5 s	Hold tandem stance for 10 s	Static balance, postural control
BESTest I: Biomechanical Constraints	Impaired base of support and CoM alignment	Improve score to 60%	Achieve score of 80%	Ankle and hip strength, flexibility
BESTest II: Stability Limits/Verticality	Moderate impairment in functional stability limits	Improve score to 75%	Achieve score of 90%	Limits of stability, postural verticality
BESTest III: Anticipatory Postural Adjustments	Difficulty with anticipatory balance reactions	Improve score to 65%	Achieve score of 85%	Anticipatory balance control
BESTest IV: Postural Responses	Impaired reactive balance control	Improve score to 55%	Achieve score of 75%	Reactive postural control
BESTest V: Sensory Orientation	Moderate impairment in sensory integration for balance	Improve score to 80%	Achieve score of 90%	Sensory integration, balance in altered sensory conditions
BESTest VI: Stability in Gait	Impaired dynamic balance during gait	Improve score to 70%	Achieve score of 85%	Dynamic balance, gait stability
BESTest (Total Score)	Overall balance impairment across multiple systems	Improve total BESTest score to 65%	Achieve total BESTest score of 80%	Comprehensive balance control
Gait Speed	Slow gait speed, associated with increased fall risk	Increase gait speed to 0.9 m/s	Achieve normal gait speed of 1.0 m/s or greater	Gait speed, overall mobility
30-second Sit-to-Stand Test	Below average lower body strength for age and gender	Increase to 10 repetitions	Achieve 12 repetitions (average for age and gender)	Lower body strength, functional mobility
Stair navigation assessment	Difficulty with stair navigation, increasing fall risk at home entrance	Ascend/descend 5 steps with handrail in 15 s	Safely navigate home stairs without handrail support	Stair mobility, lower body strength, balance
Floor transfer assessment	Risk of prolonged floor time after a fall; impacts gardening goal	Get up from floor using furniture for support	Independently transfer from floor to standing	Floor transfer ability, lower body strength, flexibility
Home safety evaluation	Multiple environmental fall risk factors present	Eliminate 3 major hazards	Implement all recommended home modifications	Environmental fall risk factors
Blood pressure check	Orthostatic hypotension contributing to fall risk	Educate on strategies to manage orthostatic hypotension	Demonstrate consistent use of strategies to prevent symptomatic BP drops	Orthostatic hypotension, dizziness
Vision screening	Visual impairment potentially contributing to fall risk	Schedule comprehensive eye exam	Obtain and consistently use appropriate vision correction	Visual acuity, depth perception
Lower extremity strength testing	Muscle weakness contributing to balance and gait issues	Improve hip abductor and ankle dorsiflexor strength to 4+/5	Achieve 5/5 strength in all lower extremity muscle groups	Muscle strength, particularly in hip and ankle
Activities-Specific Balance Confidence (ABC) Scale	Low balance confidence affecting activity participation	Increase ABC score to 75%	Achieve ABC score of 85% or higher	Balance confidence, activity participation

**Table 2:** Eight-week progressive multifactorial exercise plan with evidence-based FITT (Frequency, Intensity, Time, Type) Principles.

Week.	Exercise	Goal	Functional Goal	Frequency	Intensity	Time	Type	Progression	Supervision
1	Seated leg extensions [45,47]	Strengthen quadriceps	Improve sit-to-stand	3×/week	2 sets of 10 reps	10 min	Strength	Add resistance or reps	Initially supervised; then, independent
	Seated marching [48]	Improve hip flexor strength	Enhance gait initiation	3×/week	2 sets of 15 reps	10 min	Strength	Increase duration or speed	Initially supervised; then, independent
	Ankle pumps [45]	Increase ankle dorsiflexion strength	Improve gait clearance	Daily	3 sets of 15 reps	5 min	Strength	Increase reps or resistance	Independent
	Sit-to-stand practice [49]	Improve lower body strength	Enhance transfer ability	2×/day	5 repetitions	5 min	Functional	Increase repetitions or remove hand support	Initially supervised; then, independent
	Static balance (feet together) [50]	Improve static balance	Reduce fall risk	Daily	Hold for 30 s	5 min	Balance	Increase hold time or narrow stance	Initially supervised; then, independent
2	Standing hip abduction [47]	Strengthen hip abductors	Improve lateral stability	3×/week	2 sets of 10 reps	10 min	Strength	Add resistance band	Initially supervised; then, independent
	Calf raises [51]	Strengthen ankle plantar flexors	Enhance stair climbing	3×/week	2 sets of 15 reps	10 min	Strength	Progress to single-leg raises	Initially supervised; then, independent
	Tandem stance [45]	Improve static balance	Enhance postural control	Daily	Hold for 10 s	5 min	Balance	Increase hold time	Independent
	Walking with directional changes	Improve dynamic balance	Enhance gait adaptability	3×/week	5 min continuous	5 min	Gait	Increase complexity and speed	Supervised
	Stair stepping (with support)	Improve lower extremity strength	Safer stair navigation	2×/day	10 steps up/down	5 min	Functional	Reduce use of hand support	Supervised
3	Mini squats [48]	Strengthen lower extremities	Improve sit-to-stand and transfers	3×/week	2 sets of 10 reps	10 min	Strength	Deepen squat	Initially supervised; then, independent
	Standing leg curls	Strengthen hamstrings	Enhance gait stability	3×/week	2 sets of 10 reps	10 min	Strength	Add resistance band	Initially supervised; then, independent
	Single leg stance [50]	Improve static balance	Reduce fall risk	Daily	Hold for 10 s each leg	5 min	Balance	Increase hold time	Independent
	Obstacle course walking [52]	Improve dynamic balance	Enhance environmental navigation	3×/week	10 min continuous	10 min	Gait	Increase obstacle complexity	Supervised
	Sit-to-stand without upper extremity support [49]	Improve transfer independence	Enhance functional mobility	3×/day	8 repetitions	5 min	Functional	Increase repetitions	Initially supervised; then, independent
4	Step-ups	Strengthen lower extremities	Improve stair climbing	3×/week	2 sets of 10 reps each leg	15 min	Strength	Increase step height	Initially supervised; then, independent
	Resistance band hip abduction [47]	Increase hip abductor strength	Enhance lateral stability	3×/week	2 sets of 12 reps	10 min	Strength	Increase resistance	Initially supervised; then, independent
	Tandem walk [53]	Improve dynamic balance	Enhance gait stability	Daily	20 steps forward/backward	5 min	Balance	Add head turns or unstable surfaces	Independent
	Dual-task walking (counting backwards) [54]	Improve cognitive-motor function	Enhance safe community mobility	3×/week	10 min continuous	10 min	Gait	Increase cognitive complexity	Supervised
	Floor-to-stand transfer practice	Improve lower body strength and technique	Support gardening activities	2×/day	3 repetitions	10 min	Functional	Increase repetitions	Initially supervised; then, independent
5	Lunge exercises [55]	Strengthen lower extremities	Improve gait and stair climbing	3×/week	2 sets of 8 reps each leg	15 min	Strength	Deepen lunge or add weights	Initially supervised; then, independent
	Heel-to-toe raises	Improve ankle strength and balance	Enhance postural control	Daily	2 sets of 10 reps	5 min	Strength/Balance	Increase repetitions	Independent
	Standing on foam surface [56]	Challenge balance systems	Improve adaptation to unstable surfaces	3×/week	Hold for 30 s, 3 reps	5 min	Balance	Progress to eyes closed	Supervised
	Brisk walking [47]	Increase gait speed and endurance	Improve community mobility	5×/week	Moderate intensity	15 min	Gait	Increase speed or distance	Independent
	Stair climbing without handrail support	Improve stair navigation independence	Enhance home and community mobility	3×/week	1 flight up/down	5 min	Functional	Add flights or reduce rests	Initially supervised; then, independent
6	Single-leg mini squats	Advanced lower extremity strengthening	Improve overall functional strength	3×/week	2 sets of 8 reps each leg	15 min	Strength	Add resistance	Initially supervised; then, independent
	Resistance band ankle exercises (all directions) [47]	Comprehensive ankle strengthening	Enhance gait and balance	3×/week	2 sets of 12 reps each direction	15 min	Strength	Increase band resistance	Initially supervised; then, independent
	Tandem stance with head turns	Challenge vestibular system	Improve balance in dynamic situations	Daily	Hold for 15 s, 3 reps	5 min	Balance	Add perturbations	Supervised
	Outdoor walking with terrain changes	Improve environmental adaptation	Enhance community mobility confidence	3×/week	15 min continuous	15 min	Gait	Increase terrain difficulty	Independent
	Simulated gardening tasks	Functional strengthening and balance	Achieve gardening-related goals	2×/week	15 min continuous	15 min	Functional	Increase task complexity	Supervised
7	Obstacle course increasing difficulty	Same as previous	Achieve functional mobility	2×/week	15 continuous	15 min	Functional	Increase complexity, decrease rest breaks	Supervised
	Dual-tasking	Same as previous	Achieve functional mobility	2×/week	15 continuous	15 min	Functional	Increase dual-task challenge	Supervised
8	Obstacle course increasing difficulty	Same as previous	Achieve functional mobility	2×/week	15 continuous	15 min	Functional	Increase obstacle complexity	Supervised
	Dual-tasking	Same as previous	Achieve functional mobility	2×/week	15 continuous	15 min	Functional	Increase dual-task difficulty	Supervised

**Table 3:** Effect size comparison of assessment measures.

Assessment Measure	Initial Score	Post Score	% Change	MCID	Cohen’s *d*	Effect Size Interpretation
Timed Up and Go (TUG)	15.0 s	11.0 s	−26.67%	3.0 s	1.60	Large
Gait Speed	0.8 m/s	1.0 m/s	+25.00%	0.1 m/s	1.30	Large
30-Second Sit-to-Stand	8 reps	12 reps	+50.00%	2 reps	2.00	Large
BESTest Overall	52%	78%	+50.00%	10%	4.00	Very Large
Activities-Specific Balance Confidence (ABC)	65%	82%	+26.15%	14%	2.43	Large
Falls Efficacy Scale—International (FES-I)	35	26	−25.71%	6 points	2.25	Large

Note: For TUG and FES-I, negative percentage changes indicate improvement (lower values are better); for all other measures, positive percentage changes indicate improvement (higher values are better). Effect size interpretation: Small (*d* = 0.2), Medium (*d* = 0.5), Large (*d* = 0.8), Very Large (*d* > 1.2).

## Data Availability

Ethical and privacy constraints prevent full data disclosure.
